# Editorial: Molecular Perspectives for Plant Autophagy Regulation

**DOI:** 10.3389/fpls.2022.967916

**Published:** 2022-07-06

**Authors:** Yan Bao, Caiji Gao, Tamar Avin-Wittenberg, Jie Zhou, Faqiang Li

**Affiliations:** ^1^Shanghai Collaborative Innovation Center of Agri-Seeds, Joint Center for Single Cell Biology, School of Agriculture and Biology, Shanghai Jiao Tong University, Shanghai, China; ^2^Guangdong Provincial Key Laboratory of Biotechnology for Plant Development, School of Life Sciences, South China Normal University, Guangzhou, China; ^3^Department of Plant and Environmental Sciences, The Alexander Silberman Institute of Life Sciences, The Hebrew University of Jerusalem, Jerusalem, Israel; ^4^Department of Horticulture, Zhejiang University, Hangzhou, China; ^5^Guangdong Provincial Key Laboratory of Protein Function and Regulation in Agricultural Organisms, College of Life Sciences, South China Agricultural University, Guangzhou, China

**Keywords:** autophagy, vacuole, interaction, *ATG*, stress

Mobility and migration of plants are highly restricted. To maintain cellular homeostasis, plants have to coordinate various endogenous and external signals, with autophagy playing a critical role in those processes. Autophagy is a programmed cellular mechanism that can deliver unwanted targets (such as protein aggregates, damaged organelles, virus and pathogens) for degradation in the lytic organelle (the vacuole in plants). In addition to sustaining a basal low level of autophagy for plant growth, different environmental stresses, biotic (pathogen or virus infection etc.), or abiotic (salt, drought, oxidation etc.), can trigger autophagy induction to counteract stresses for survival.

In the scope of this Research Topic, Wang et al. comprehensively reviewed the mechanism, activation, and signaling pathways of autophagy in plants. The authors introduced the three major types of autophagy found in plants, namely microautophagy, macroautophagy, and megaautophagy, and their mechanisms. Among them, macroautophagy, referred to as autophagy from here on, is the most described and well-studied. The authors went on to describe the activation of plant autophagy, mainly the initiation of autophagy by Target of Rapamycin (TOR) kinase, in greater detail.

Autophagy was shown to be induced by nutrient starvation. The manuscript describes the various nutrients whose deprivation induces autophagy, such as nitrogen, carbon, sulfur, and phosphorus. Autophagy also plays a vital role in response to biological factors such as pathogens or viruses that adversely affect the growth of plants, which has been described well in this review. On the one hand, autophagy can promote the hypersensitive response (HR) to prevent the invasion of pathogens. On the other hand, autophagy is also affected by the pathogens, thereby facilitating their invasion.

Autophagy is also induced by plant abiotic stress. The authors introduced the adverse effects of various stresses, providing recent examples of the role of autophagy in plant stress tolerance. In the last part of the review, the authors made great efforts to describe the regulation of autophagy by hormonal signals such as abscisic acid, ethylene, brassinosteroids, auxin, and jasmonic acid, which can indeed induce the autophagy to help plants cope with stress. Overall, the authors reviewed many studies on autophagy, thus conveying essential information to researchers and exploring the content of follow-up research.

The underlying mechanism of microautophagy remains largely unknown in most organisms, especially in plants. During maize endosperm development, the main storage proteins prolamins in starchy endosperm cells accumulate directly in protein bodies within the endoplasmic reticulum (ER). In contrast, they are transported to vacuoles *via* an autophagic route in adjacent aleurone cells. In this Research Topic, Ding et al. showed that prolamins were directly taken up by vacuoles in aleurone cells *via* a process resembling microautophagy. They further identified 143 candidate proteins involved in microautopahgy in aleurone cells by transcriptomic and proteomic approaches. Two listed proteins, namely phospholipase-Dα5 and a putative Euonymus lectin (EUL)-related protein were shown to alter tonoplast morphology and promote vacuolar invagination when overexpressed in *Arabidopsis thaliana* leaf protoplasts. They also accumulate specifically at the tonoplast surfaces involved in the engulfment of protein bodies, thus implying their potential roles in membrane modification and/or microautophagy. This study represents an important contribution for future dissecting the mechanism of microautophagy in plants.

Selective autophagy is an important mechanism that helps selectively remove cellular components such as protein aggregates or damaged organelles through the autophagy pathway. In this process, autophagy receptors containing ATG8-interacting motif (AIM) or ubiquitin-interacting motif (UIM) can bind to both the cargo molecules and ATG8, thereby facilitating autophagosome formation or recruitment of autophagy substrates. Compared with selective autophagy research in mammalian and yeast systems, studies regarding the autophagy receptors involved in the regulation of selective autophagy in plants are more scarce. In this Research Topic, Liu et al. comprehensively summarized and classified the AIM or UIM motif-containing proteins in plants. More importantly, they discussed the conserved and diversified functions of these proteins in the regulation of various types of selective autophagy and other biological processes. The manuscript provides a sound basis for the future functional study of AIM or UIM motif-containing proteins in plants.

As mentioned above, autophagy has been extensively studied in plant-microbe interactions. However, from the perspective of microbes, the role of autophagy in their host colonization is less touched. Shiraishi and Sakai discussed the strategies (with a particular focus on autophagy) that eukaryotic microbes leverage to propagate in the phyllosphere. *Candida boidinii* is a methylotrophic yeast that can live and grow on the leaf surface of Arabidopsis. Using this unique system, it was found that daily methanol fluctuation in the phyllosphere was critical for pathogen survival. In line with this finding, studies in *Komagataella phaffii* showed that a family of Wsc family proteins could sense the levels of extracellular methanol and regulate pexophagy in a negative manner. In addition to autophagy, the Cvt (cytoplasm-to-vacuole targeting) pathway was also found to be required for yeast nitrogen homeostasis. The underlying mechanisms regarding the interactions between Cvt and autophagy in plant-microbe interactions warrant further investigation.

As autophagy has been shown to impact plant growth and yield, specifically during biotic and abiotic stress, it has been postulated that manipulating autophagy may positively affect plant growth under field conditions. In their review manuscript featured as part of this research topic, Thanthrige et al. summarize the current knowledge regarding the role of autophagy in plant stress response, particularly focusing on stresses encountered under field conditions. They also touch upon the function of autophagy in nutrient remobilization, also affecting plant growth and yield. Interestingly, the authors highlight the possible role of autophagy in plant-symbiont interaction, a fascinating research topic that should be further explored.

The authors go on to describe the possible ways in which autophagy can be manipulated in crops to improve plant stress tolerance. Transgenic over-expression of *ATG* genes has been shown to alleviate abiotic stress in many plant species and possibly promote pathogen resistance. In addition, several transcription factors were shown to influence autophagy activation. These can also be considered as targets for autophagy enhancement. Apart from its activation for increased stress tolerance, autophagy can also be used to improve nutrient remobilization to seeds and biofuel crops, specifically under non-favorable conditions. Although transgenic approaches demonstrate promising results, non-tersgenic approaches for autophagy induction are also being sought out. The authors describe the current efforts made today and point to interesting new avenues to be explored in that field.

Finally, the authors raise the issue of selective autophagy and crop improvement. Until now, studies focused on enhancing general autophagic flux, rather than focusing on the selective degradation of stress-specific targets. Modulating selective autophagy may confer higher precision to autophagy modulation, circumventing trade-offs between growth and stress response. The authors point to several exciting directions to be explored regarding plant autophagy and crop improvement in the following years.

## Author Contributions

All authors contributed to the drafting of this Editorial. YB and TA-W revised the Editorial. YB, CG, TA-W, JZ, and FL approved it for publication. All authors contributed to the article and approved the submitted version.

## Funding

Research in the lab of YB was supported by the National Natural Science Foundation of China (32170282) and China Agriculture Research System of MOF and MARA. Research in the lab of CG was supported by grants from the National Natural Science Foundation of China (32061160467 and 31870171). Research in the lab of TA-W was supported by the Israel Science Foundation (ISF), grant number 1942/19. Research in the lab of JZ was supported by the Fundamental Research Funds for the Central Universities (226-2022-00122). Research in the lab of FL was supported by National Natural Science Foundation of China (31970307).

## Conflict of Interest

The authors declare that the research was conducted in the absence of any commercial or financial relationships that could be construed as a potential conflict of interest.

## Publisher's Note

All claims expressed in this article are solely those of the authors and do not necessarily represent those of their affiliated organizations, or those of the publisher, the editors and the reviewers. Any product that may be evaluated in this article, or claim that may be made by its manufacturer, is not guaranteed or endorsed by the publisher.

